# Fulminant Phlegmonitis of the Esophagus and Stomach

**DOI:** 10.7759/cureus.77772

**Published:** 2025-01-21

**Authors:** Jiaming Lei, Ling Wu

**Affiliations:** 1 Department of Gastroenterology, People’s Hospital of Leshan, Leshan, CHN; 2 Department of Cardiology, The Affiliated Hospital of Southwest Medical University, Luzhou, CHN

**Keywords:** esophagus, fever, infection, phlegmonous gastritis, vomiting

## Abstract

Phlegmonous esophagogastritis is a rare and life-threatening condition characterized by purulent inflammation of the submucosal and muscularis layers of the esophagus and stomach. We report the case of a 59-year-old male with a history of hypertension and prior herbicide ingestion who presented with fever, sore throat, chest pain, and progressive abdominal pain. Initial misdiagnosis of pancreatitis delayed treatment. Endoscopy revealed extensive mucosal detachment, submucosal edema, purulent exudates, and fistula formation, while computed tomography (CT) demonstrated gastric wall thickening and intramural gas. Microbiological analysis identified *Streptococcus* spp., Epstein-Barr virus, and cytomegalovirus co-infections. Despite intensive therapy, including antibiotics, antivirals, nutritional support, and pleural drainage, the patient’s condition deteriorated, leading to multi-organ failure and death. This case highlights the diagnostic challenges posed by nonspecific symptoms of phlegmonous esophagogastritis, the critical importance of early endoscopic evaluation, and the value of microbiological analysis for tailored treatment. Early recognition and timely intervention are essential to improving outcomes for this potentially fatal condition.

## Introduction

Esophageal and gastric diseases are well known for their high prevalence; however, most of these conditions primarily affect the mucosal layer, while diseases involving the submucosa and muscularis layers are relatively rare. Phlegmonous esophagogastritis is a rare condition characterized by purulent inflammation of the submucosa and muscularis layers of the esophagus and stomach. Since its initial description by Cruveilhier in the 18th century, only sporadic cases have been reported, including 441 cases of phlegmonous gastritis and 35 cases of phlegmonous esophagitis [1]. The clinical manifestations of this disease are highly variable, ranging from fever, sore throat, chest pain, abdominal pain and distension, nausea, and vomiting to severe outcomes such as septic shock and death.

Historically, the diagnosis of phlegmonous esophagogastritis relied heavily on intraoperative findings or postmortem examinations. However, advancements in diagnostic imaging, including computed tomography (CT) and endoscopic techniques - particularly endoscopic ultrasound (EUS) - have significantly enhanced our understanding and identification of this rare disease [2]. Despite these advancements, there remains a lack of well-established diagnostic and management strategies for phlegmonous esophagogastritis.

Here, we present a rare yet classic case of phlegmonous esophagogastritis, showcasing the disease’s striking features through endoscopic findings. Unfortunately, in this case, delayed diagnosis led to disease progression, underscoring the critical need for improved recognition and timely management.

## Case presentation

A 59-year-old male patient with a history of hypertension and herbicide ingestion (specific agent unknown) four years prior presented with a sore throat that developed 10 days before admission. Within two days, the symptoms progressed to severe chest pain and upper abdominal pain, accompanied by nausea, vomiting of gastric contents, fever (maximum temperature 40°C), cough with yellow sputum, and dyspnea. There was no hematemesis, diarrhea, melena, or hematochezia. Physical examination revealed diminished breath sounds in the right lung, abdominal distension, and localized muscle tension in the upper abdomen.

The laboratory tests are shown in Table 1. Laboratory tests indicated bacterial infection predominantly involving neutrophils, with significant elevations in procalcitonin and C-reactive protein. Arterial blood gas analysis showed reduced partial oxygen pressure, hypoalbuminemia, and abnormal liver function, consistent with severe infection. Additionally, elevated blood amylase (267 U/L) and lipase (636.1 U/L) levels were noted.

**Table 1:** Patient's laboratory test items.

Laboratory Investigations	Parameters	Patient Values	Units of Measurement	Reference Range
Complete Blood Count (CBC)	White Blood Cell	18.17	×10⁹/L	4-10
	Neutrophils	13.99	×10⁹/L	2-8
	Red Blood Cell	4.33	×10⁹/L	4-10
	Hemoglobin	117	g/L	>130
	Platelets	211	×10⁹/L	100-300
Arterial Blood Gas	PH	7.479	-	7.35-7.55
	PO₂	65	mmHg	>70
	PCO₂	28.0	mmHg	<40
Biochemical Indexes	Alanine aminotransferase (ALT)	281	U/L	<50
	Aspartate aminotransferase (AST)	268	U/L	<35
	Albumin	23.3	g/L	>40
	Total Protein	49.9	g/L	>60
	Urea	24.89	mmol/L	<5
	Creatinine	128	µmol/L	<130
	Total Bilirubin	47.3	µmol/L	<17.1
	Serum Amylase	267	U/L	<75
	Triglycerides	1.54	mmol/L	0.45–1.7
	Total Cholesterol	1.96	mmol/L	< 5.2
	Potassium K	3.1	mmol/L	3.5-5.5
	Sodium Na	138.5	mmol/L	135-155
Tumor Marker	Cancer Antigen 125	68.1	U/mL	<20
Index of Infection	Procalcitonin	53.92	ng/mL	<0.01
	C-reactive Protein	189	mg/L	<10
	Blood Culture	-	/	-
Secretions of Next-Generation Sequencing (NGS)	Streptococcus anginosus	+	/	-
	Epstein-Barr virus	+	/	-
	Cytomegalovirus	+	/	-

**Figure 1:**
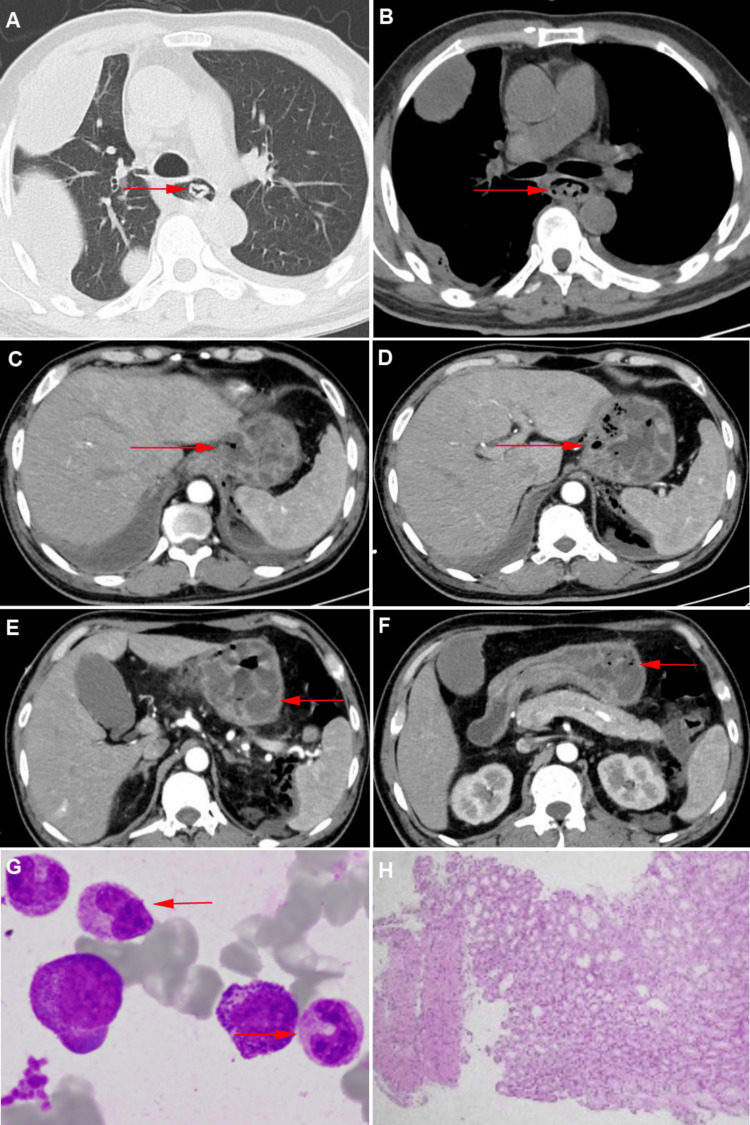
This figure illustrates the CT imaging characteristics, bone marrow smear, and pathology (indicated by red arrows). A. Esophagus (lung window); B. Esophagus (mediastinal window); C. Gastroesophageal junction; D. Upper gastric body; E. Middle gastric body; F. Gastric antrum; G. Bone marrow smear; H. Pathological tissue section of gastric antrum mucosa biopsy.

Contrast-enhanced CT revealed massive right pleural effusion, esophageal dilation, and thickening of the mid-to-lower esophageal wall with intraluminal mixed-density material. The gastric wall was markedly edematous and thickened, with intramural gas suggestive of phlegmonous inflammation (Figures 1A-1F). Based on these findings, the differential diagnosis was expanded to include gastrointestinal perforation, necrotizing gastritis, and phlegmonous esophagogastritis. The presence of intramural gas and significant wall thickening prompted consideration of infectious causes affecting the submucosal and muscularis layers.

**Figure 2:**
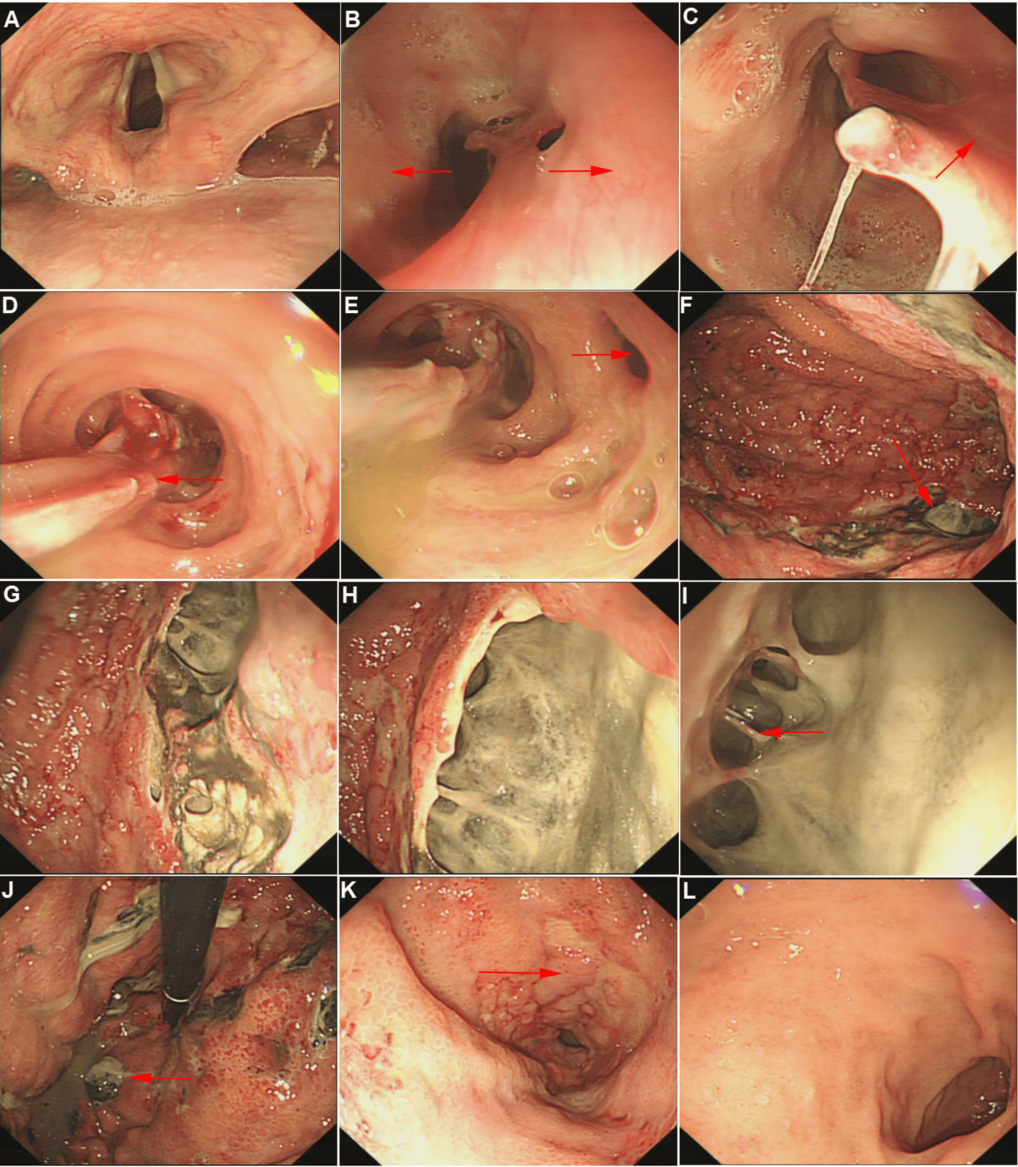
This figure depicts the endoscopic findings of the esophagus and stomach, highlighting the visual manifestations. A. Pharynx; B. Esophageal inlet (right red arrow indicates normal mucosa; left red arrow indicates mucosal detachment); C. Upper esophagus (red arrow shows esophageal lumen narrowing); D. Esophageal mucosal contraction; E. Esophageal fistula (red arrow); F. Gastric body (distant view); G, H. Gastric body (close-up views of lesions); I. Bridging blood vessels (red arrow); J. Gastric fundus (red arrow suggests potential perforation); K. Gastric antrum; L. Duodenum.

Upper gastrointestinal endoscopy demonstrated multilayered mucosal changes in the upper esophagus with severe mucosal disruption. The middle esophagus extending to the gastroesophageal junction exhibited circumferential mucosal detachment and extensive purulent exudates, with evidence of fistula formation. The gastric mucosa appeared dark red, ischemic, and edematous, with multiple purulent deposits in the gastric fundus. A large ulcerative lesion with purulent exudates and exposed submucosal vessels was identified on the posterior wall of the greater curvature. A biopsy was performed on a safe area along the lesser curvature to minimize bleeding. Given the extensive mucosal damage, the likelihood of a bacterial etiology was considered high, and biopsies were obtained from a relatively intact area along the lesser curvature to minimize bleeding risk. The duodenum appeared normal (Figure 2).

Thoracentesis yielded yellow, turbid pleural fluid consistent with exudative effusion. Bone marrow aspiration showed hyperactive myelopoiesis, with 71.5% granulocytes and prominent cytoplasmic granulation, indicating infection (Figure 1G). Pathological analysis of the endoscopic biopsy revealed chronic active inflammation with focal neutrophilic infiltration, though limited by the biopsy depth (Figure 1H). Purulent gastric secretions underwent next-generation sequencing (NGS), which identified *Streptococcus* spp., Epstein-Barr virus (EBV), and cytomegalovirus (CMV) as co-infecting pathogens (Table 1).

This combination of findings, including purulent inflammation confirmed by endoscopy, imaging, and microbiological results, supported the diagnosis of acute phlegmonous gastritis with localized perforation. Treatment included oxygen therapy, fasting, intravenous antibiotics (meropenem 1 g every eight hours), antiviral therapy (ganciclovir 0.25 g every 12 hours), acid suppression (esomeprazole 40 mg every 12 hours), human albumin infusion (20 g daily), parenteral nutritional support, and pleural drainage to re-expand the lung. Aggressive fluid resuscitation was also administered.

Despite intensive treatment, the patient’s condition continued to deteriorate due to the extensive gastric perforation, severe infection, and widespread disease involvement. The patient declined further surgical intervention due to financial constraints and ultimately succumbed to multi-organ failure secondary to septic shock.

## Discussion

In this case, the initial diagnosis and treatment were directed toward pancreatitis due to symptoms such as abdominal pain, fever, dyspnea, elevated amylase levels, and CT findings suggestive of peripancreatic exudation. The definitive diagnosis of phlegmonous esophagogastritis was only established after endoscopy, leading to a delay in targeted treatment. This delay may have contributed to disease progression, although the rapid deterioration of the patient’s condition was closely related to the extensive disease involvement. Phlegmonous esophagogastritis can result in numerous complications, including peritonitis, mediastinitis, empyema, perforation, sepsis, and even death.

Phlegmonous esophagogastritis is characterized by purulent bacterial infection and significant thickening of the gastric wall, especially in the submucosal layer. It can arise from localized or disseminated infections and may involve part or the entirety of the stomach. In some cases, the esophagus and intestines are also affected. Although the pathogenesis remains unclear, mucosal injury, intoxication, hypochlorhydria, immunosuppression, and coexisting diseases, such as malignancies, infections, or connective tissue disorders, are considered potential risk factors [3]. However, approximately 50% of patients have no prior significant health issues [4]. This patient had a history of herbicide ingestion several years ago, which may have caused damage to the gastric and esophageal mucosa. The current illness began with a viral infection, presenting as fever and sore throat, followed by rapid disease progression, resembling a positive feedback loop leading to digestive tract involvement.

The microbial composition in phlegmonous esophagogastritis is often complex, involving bacterial and viral agents that cause fatal damage after disruption of the gastrointestinal mucosal barrier and altered microbiota. Streptococcal infections, particularly β-hemolytic Group A *Streptococcus*, account for 70%-75% of cases and are the most commonly associated pathogens in fatal cases [5]. Different microorganisms produce distinct clinical presentations, such as phlegmonous, emphysematous, or necrotizing gastritis. In this case, purulent secretions analyzed by NGS confirmed *Streptococcus* infection, along with EBV and CMV co-infections, corroborating these epidemiological trends. The study reported that in the pre-antibiotic era, mortality rates for this condition were as high as 92%. With the advent of antibiotics, overall mortality has decreased to 42%, with localized infections having a mortality rate of 17%, while diffuse disease still carries a mortality rate of up to 60% [6].

CT imaging typically shows esophageal and gastric wall thickening, hypodensity, and layered enhancement after contrast administration. Gas-producing bacterial infections may produce intramural gas. Endoscopy reveals extensive ischemic changes and necrosis of the mucosa. In this patient, endoscopy allowed clear visualization of the submucosal layer through the ulcer surface (likely formed by spontaneous decompression), along with retained purulent material. Endoscopic ultrasound (EUS) revealed diffuse thickening of the submucosal layer with hypoechoic lesions within the submucosa. While primarily involving the submucosal layer, the inflammation can extend to all layers of the gastric wall. However, CT findings alone are insufficient for a definitive diagnosis and can only provide supportive evidence. EUS is superior to CT in evaluating gastric wall thickness and the extent of inflammation. Histopathologically, the submucosal and muscularis layers exhibit marked edema and infiltration by neutrophils and plasma cells, along with intramural hemorrhage, necrosis, and submucosal vascular thrombosis. In this case, biopsies of relatively normal mucosa revealed chronic active inflammation, but standard forceps biopsies often fail to obtain sufficient submucosal tissue. Studies suggest that obtaining submucosal tissue with endoscopic resection or snare biopsies may improve diagnostic accuracy [7]. In cases of transmural infection, culture and mucosal biopsies may facilitate diagnosis.

Conservative antibiotic therapy remains the mainstay of treatment, although studies suggest that surgical resection of the stomach is associated with reduced mortality compared to medical therapy alone [8]. In addition to traditional open surgery, endoscopic drainage has been proposed as a viable treatment for phlegmonous esophagitis [9]. Several studies have also described the benefits of jejunostomy or gastrostomy for early enteral feeding, which can improve clinical outcomes [10]. Data on esophageal stricture as a late complication of phlegmonous esophagitis remain limited. In this case, severe complications, such as gastric perforation and esophagopleural fistula, posed significant surgical risks, and the patient’s prognosis was poor despite aggressive treatment. Without early diagnosis and appropriate management, the condition carries a high mortality rate, underscoring the importance of early recognition and intervention.

In summary, we report a rapidly progressive and fatal case of diffuse phlegmonous esophagogastritis with clear documentation of late-stage mucosal manifestations in the esophagus and stomach. The nonspecific clinical symptoms pose diagnostic challenges, highlighting the need for increased awareness of this rare and often lethal condition.

## Conclusions

In this field, controversy exists regarding the optimal management approach, particularly in the balance between conservative medical treatment and the role of early surgical intervention. While some studies suggest that surgical resection can significantly improve survival in patients with diffuse involvement or complications like perforation, others advocate for aggressive medical therapy as the first-line treatment in localized cases. Additionally, the absence of standardized diagnostic criteria and limited data from large-scale studies contribute to ongoing debates about the best practices for early recognition and intervention. Recent advancements in diagnostic methodologies represent significant achievements in this field. The integration of NGS for microbiological analysis has enhanced pathogen identification, providing insights into polymicrobial infections and viral co-pathogens, as demonstrated in this case.

Further research is urgently needed to address these controversies and to establish more effective diagnostic and management strategies, especially given the high mortality rate associated with this condition. Collaborative efforts to develop evidence-based guidelines could significantly improve clinical outcomes in the future.
